# Editorial: Natural products in the treatment of neurological diseases: identification of novel active compounds and therapeutic targets

**DOI:** 10.3389/fphar.2023.1294625

**Published:** 2023-09-22

**Authors:** Jia Zhao, Di Wang, Wei Cui, Hansen Chen

**Affiliations:** ^1^ School of Chinese Medicine, The University of Hong Kong, Hong Kong, SAR, China; ^2^ Engineering Research Center of Chinese Ministry of Education for Edible and Medicinal Fungi, School of Plant Protection, Jilin Agricultural University, Jilin, China; ^3^ Department of Physiology and Pharmacology, Ningbo University, Ningbo, China; ^4^ Department of Neurosurgery, Stanford University School of Medicine, Stanford, CA, United States

**Keywords:** natural products, neurological diseases, stroke, epilepsy, depression, therapeutic targets

Stroke, epilepsy, and depression rank among the most prevalent neurological and psychiatric disorders, yet viable treatment options remain limited (Campbell and Khatri, 2020; Herrman et al., 2022; Asadi-Pooya et al., 2023). This Research Topic focuses on the potential of natural products to address these disorders and highlights the discovery of these products and their novel therapeutic targets.

Currently, the only FDA-approved treatments for ischemic stroke are thrombectomy and tissue plasminogen activator. However, these treatments have a limited therapeutic window and carry an increased risk of hemorrhagic transformation (Tsivgoulis et al., 2023; Widimsky et al., 2023). In this Research Topic, Heng Cai et al. obtained a novel exosome-like nanoparticles from *Momordica charantia* entitled MC-ELNs, which show protection against ischemic stroke. The characterization of MC-ELNs was systematically analyzed. In transient cerebral ischemic rats, MC-ELNs promotes neuronal survival to inhibit cerebral ischemia, shows capacity of crossing blood-brain barrier (BBB) and further protects the integrity of BBB. The authors also confirm that these effects of MC-ELNs are related to its inhibition on MMP-9 activation and suppression on neuronal apoptosis via the regulation of serine/threonine kinase/glycogen synthase kinase 3β signalling. This study provides a possibility on the application of plant exosome-like nanoparticles for ischemic stroke treatment.

Post-stroke depression (PSD) affects approximately one-third of stroke survivors. In this context, natural products with demonstrated antidepressant properties hold great promise. Chaoyou Fang et al. critically assess the existing body of evidence concerning the efficacy of various natural products in addressing PSD. Among these products are St. John’s wort, Rhodiola rosea, Bacopa monnieri, Ginkgo biloba, and omega-3 fatty acids. The synthesis of available research suggests that natural products hold promise as a viable and safe avenue for PSD treatment. Nonetheless, it is imperative to underscore the necessity for further extensive research to definitively establish both the efficacy and safety of these interventions.

The COVID-19 pandemic has exacerbated the global prevalence of major depressive disorder by 27.6% (COVID-19 Mental Disorders Collaborators, 2021). Selective serotonin reuptake inhibitors including citalopram are primary treatments for major depressive disorder but are hindered by potential sexual and gastrointestinal side effects (Park and Zarate, 2019). Traditional Chinese Medicine (TCM) was used for the treatment of depression from ancient times to the modern era. It is necessary to understand the antidepressant effects and molecular mechanism of active compounds from TCM (Li et al., 2020). In this Research Topic, Li et al. provided a review of the commonly used animal model of depression and antidepressant activity and the mechanism of active components in Panax Notoginseng.

One-third of patients continue to experience drug-resistant epilepsy despite the availability of approximately 30 anti-epileptic drugs. It is crucial to identify new anti-epileptic drugs for the treatment of epilepsy (Zheng et al., 2021). Wang et al. identified aloesone from Aloe vera with network pharmacology. Aloesone could treat seizures by activating c-SRC in a rat model of epilepsy. This study provided a potential compound for the treatment of seizures.

Neurological disorders often share neuroinflammatory response mechanisms, making neuroinflammation a key target for treatment. Shuai Wang and Xin Qi conducted a comprehensive review of the potential of astaxanthin, a natural compound, in modulating neuroinflammation. This paper highlights astaxanthin’s remarkable anti-oxidative stress properties and its ability to combat inflammation, targeting key pathways such as nuclear factor κB and mitogen-actived protein kinase pathways. The disruption of BBB integrity and peripheral inflammation are two significant factors contributing to neuroinflammation. The paper underscores astaxanthin’s role in preserving the integrity of the BBB in stroke models, a critical aspect of maintaining brain homeostasis. Astaxanthin has also demonstrated the capacity to mitigate peripheral inflammation across various disease models, which, in turn, can contribute to BBB protection. With these protective effects, astaxanthin has shown promise in safeguarding against multiple neurological disorders, including Alzheimer’s disease, Parkinson’s disease, depression, cardiac-cerebral vascular diseases, spinal cord injury, epilepsy, and diabetes-induced neuropathy. Nonetheless, the authors stress the importance of additional research to comprehensively elucidate the mechanisms of action of astaxanthin and fully realize its therapeutic potential.

Lastly, natural products have the potential to complement stem cell therapies, offering solutions for traditional treatments-resistant disorders. However, a low transplantation and survival rate for transplanted stem cells largely impeded its clinical applications. In this Research Topic, Guo et al. and Wei et al. have superlatively summarized the underlying mechanisms of cryptotanshinone, a diterpenoid quinone found in Salvia miltiorrhiza Bunge, and baicalin, a flavonoid compound isolated from Scutellaria baicalensis Georgi on the actions of stem cell behaviour. Importantly, these authors highlighted the possibilities for improving the clinical efficacy of stem cell therapy from combining stem cell therapy with cryptotanshinone or baicalin, providing a support for the use of these natural products in the treatment of complex diseases.

In summary, this Research Topic underscores the significant promise of natural products in addressing neurological disorders and highlights the need for ongoing research and clinical exploration to fully realize their therapeutic potential.
